# Clinical application of fluorescence endoscopic imaging using hypericin for the diagnosis of human oral cavity lesions

**DOI:** 10.1038/sj.bjc.6605357

**Published:** 2009-10-06

**Authors:** P S P Thong, M Olivo, W W L Chin, R Bhuvaneswari, K Mancer, K-C Soo

**Affiliations:** 1 Division of Medical Sciences, National Cancer Centre Singapore, 11 Hospital Drive, Singapore 169610, Singapore; 2 Singapore Bioimaging Consortium, Biomedical Sciences Institutes, 11 Biopolis Way, #02-02 Helios, Singapore 138667, Singapore; 3 Laboratory Medicine Service, Changi General Hospital, 2 Simei Street 3, Singapore 529889, Singapore; 4 Department of Surgery, Singapore General Hospital, Outram Road, Singapore 169608, Singapore

**Keywords:** fluorescence diagnosis, endoscopy, image analysis, oral cancer, hypericin

## Abstract

**Background:**

Diagnosis of oral cancer is conventionally carried out using white light endoscopy and histopathology of biopsy samples. However, oral tumours are mostly superficial and the lesion and its margins can be difficult to visualise under white light. We present clinical data on fluorescence diagnostic imaging of oral lesions using hypericin, a plant-based photosensitiser.

**Methods:**

Fluorescence images of lesions and normal tissue were captured using an endoscope after hypericin administration. The images were analysed to extract their colour parameters, which, along with the red-to-blue intensity ratios, were analysed and used to discriminate between tissue types. The results were correlated with those from histopathology.

**Results:**

The red-to-blue intensity ratio increased from normal to hyperplastic to cancerous tissue and was a good parameter to discriminate between these tissue types, with sensitivity and specificity levels of 90% and above.

**Conclusion:**

Our results show that hypericin fluorescence imaging has the potential to be used for the clinical diagnosis of oral cancer. Further study to enhance the clinical potential of this technique includes the development of a real-time image processing and analysis system interfaced to the endoscope to enable same-day cancer diagnosis and demarcation of lesion margins in a clinical setting.

Cancer of the oral cavity is one of the most common forms of malignancy worldwide, with a majority of the cases occurring in men, among whom the disease ranks as the eighth most common cancer (Parkin *et al*, 2005; Garcia *et al*, 2007). South Asia, in particular, has relatively high incidence rates for both men and women (Parkin *et al*, 2005). The 5-year survival is poor (about 50%) (Garcia *et al*, 2007). Early diagnosis is the key to a good prognosis, hence it is important to develop a rapid and accurate means for the detection of early oral cancer (Sciubba, 2001; Neville and Day, 2002; D’Silva and Ward, 2007).

The predominant type (over 90%) of tumours in the oral cavity is squamous cell carcinoma (SCC) (Neville and Day, 2002; D’Silva and Ward, 2007). Diagnosis is conventionally achieved by white light endoscopy, followed by histopathological examination of biopsy tissue. However, there are a number of challenges associated with the current diagnostic approach. First, oral lesions tend to be flat and it might be difficult to distinguish benign from malignant lesions using white light endoscopy. Second, it might be difficult to determine the margins of lesions during procedures. Moreover, genetically altered cells have been found to extend into clinically and histologically ‘normal’ regions (van Houten *et al*, 2004; Poh *et al*, 2006). Third, histopathology requires time for processing. Although biopsy procedures are generally safe, they nevertheless carry a small risk of complications for patients. The physician has to balance between reducing the number of biopsies and a missed diagnosis through error in the choice of a sampling site. Thus, although histopathology remains the gold standard for a definitive diagnosis for oral cancer, the technique has its own limitations, including small sample size and artefacts arising from sampling and processing. Moreover, the interpretation of histology results is dependent on the skill and experience of trained specialists.

Fluorescence diagnostic imaging is a relatively new technique that involves the use of a photosensitising drug to visualise lesions through tissue fluorescence. Results from studies using 5-aminolevulinic acid (5-ALA)-induced protophorphyrin IX (PpIX) fluorescence have shown this technique to be superior to white light endoscopy for the diagnosis of various types of cancer, including those in the oral cavity (Betz *et al*, 2002; Zheng *et al*, 2002, 2004; Csanady *et al*, 2004). Hypericin is a plant-based photosensitiser that accumulates selectively in abnormal cells, including tumour cells (Olivo *et al*, 2006). We have previously shown the potential for hypericin to be used as a fluorescence contrast agent in *ex vivo* cytology and in *in vivo* fluorescence imaging for the diagnosis of bladder cancer (Olivo *et al*, 2003; Sim *et al*, 2005; Fu *et al*, 2007; Kah *et al*, 2008). We anticipate that the use of hypericin with fluorescence endoscopy can improve the sensitivity and specificity of oral cancer diagnosis compared with white light alone, by providing an enhanced contrast between the lesion and surrounding normal tissue. In comparing hypericin with 5-ALA-induced PpIX fluorescence cystoscopy for the detection of bladder cancer, D’Hallewin *et al* (2002) observed that hypericin fluorescence provides improved specificity and is subject to reduced photobleaching. Hypericin may thus offer similar advantages over 5-ALA-induced PpIX fluorescence for endoscopic imaging of oral cancer. In this study, we present hypericin fluorescence diagnostic imaging as a technique that can complement standard white light endoscopy for the diagnosis of oral cancer by (1) facilitating guided biopsies in the clinic, thereby reducing the number of biopsies taken; (2) providing visualisation of tumour margins during surgical procedures; and (3) providing a means for same-day diagnosis in the clinic.

## Materials and methods

### Patients

Twenty-three patients (mean age: 59.3 years, s.d.: 13.5, range: 35–80 years, 74% males) with clinically suspicious or histologically proven lesions in the oral cavity were recruited for the study after obtaining informed consent. Within this patient pool, 13 lesions were histopathologically characterised as hyperplasia, three as cellular pleomorphic adenoma of the palate, four as dysplasia and 12 as SCC. Hyperplasia lesions typically ranged in sizes between 0.5 and 1 cm, whereas SCC lesions were typically larger, ranging from 1 to 4 cm, with depths of invasion ranging from 3 to 20 mm. Ulceration was recorded in 25% of hyperplasia patients and in 55% of SCC patients. Lymph node involvement was recorded in 25% of patients with SCC. In addition to 32 lesions imaged, 31 normal oral cavity sites in patients were also imaged, making a total of 63 sites that were imaged. This study was approved by the Institutional Review Board of the National Cancer Centre Singapore.

### Hypericin preparation and administration

A stock solution was prepared by dissolving 1 mg of hypericin (Molecular Probes, Invitrogen Corporation, Carlsbad, CA, USA) in 125 *μ*l of ethanol. Hypericin stock (125 *μ*l) was added to 125 ml of 1% serum albumin in phosphate-buffered saline, and stored at −20°C until use. Just before topical application, an 8 *μ*M hypericin instillation solution was prepared by diluting the thawed preparation in saline, and then filtering. Hypericin solution (100 ml) was topically administered to each patient by oral rinsing over 30 min.

### Fluorescence endoscopy

**Figure 1:**
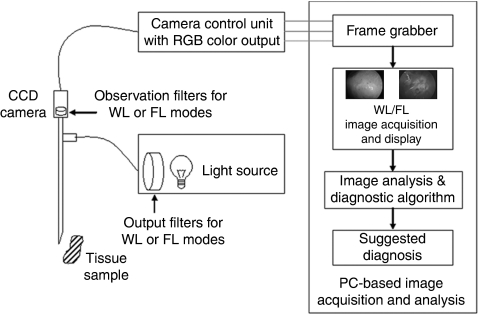
Schematic diagram of the setup showing a fluorescence endoscopy system interfaced to a PC-based image acquisition and analysis system for fluorescence diagnostic imaging of lesions in the human oral cavity in a clinical setting. WL stands for white light; FL, fluorescence.

The schematic diagram in Figure 1 shows the setup of a fluorescence endoscopy system (Karl Storz Endoscope, Karl Storz GmbH & Co., Tuttlingen, Germany) interfaced to a PC-based image acquisition and analysis system for fluorescence diagnostic imaging of human oral lesions in the clinic. The light source is a 100 W xenon short arc lamp (Karl Storz D-light system, Karl Storz GmbH & Co.) with filter options for white light (WL) and fluorescence (FL) excitation. Fluorescence excitation light is filtered by a band pass filter (380–440 nm), whereas the emitted fluorescence signal is filtered by a long pass observation filter (cut-on wavelength 450 nm). White light and fluorescence images are captured by a 3-chip CCD camera (Karl Storz Tricam SL-PDD, Karl Storz GmbH & Co.) and input to the PC-based image acquisition and analysis system.

At 1.5 to 4 h (mean 2.2 h, s.d. 0.6 h) after hypericin administration, fluorescence images of suspected lesions and normal regions in the oral cavity of patients were acquired. Corresponding white light images were acquired before the administration of hypericin and/or after the completion of fluorescence imaging to avoid photobleaching of hypericin because of white light illumination.

### Image analysis

The fluorescence images acquired were analysed to extract the red, green and blue (RGB) intensities, as well as the hue, saturation and intensity (HSI) values of the images. The red-to-blue (R/B) and red-to-green (R/G) intensity ratios were also calculated for each image. Among the images acquired for each site, three sharpest images were selected to obtain the mean values of R/B, R/G, H, S and I. These mean image parameters were used to distinguish the tissue type at three levels: (1) normal *vs* hyperplastic tissue; (2) normal *vs* SCC tissue; and (3) hyperplastic *vs* SCC lesions.

### Statistical analysis and calculations

To evaluate how well the image parameters can discriminate between tissue types, one-way ANOVA analysis with Bonferroni's multiple comparison tests was carried out using GraphPad Prism (GraphPad Software Inc., La Jolla, CA, USA) for a pairwise comparison of the means. A *P*-value of less than 0.05 is considered significant and is indicated by asterisks over the respective data pair (^*^P<0.05; ^**^P<0.01; ^***^P<0.001). The image parameters were also analysed by the Receiver Operating Characteristics (ROC) curves method using GraphPad Prism. ROC analysis provided the areas under the curve, *P*-values and cutoff values for different sensitivity and specificity levels. The sensitivity, specificity, positive predictive value (PPV) and negative predictive value (NPV) of each test were calculated using the following formulae: 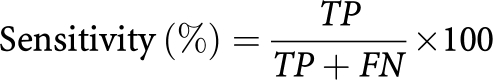


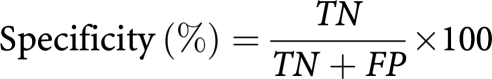


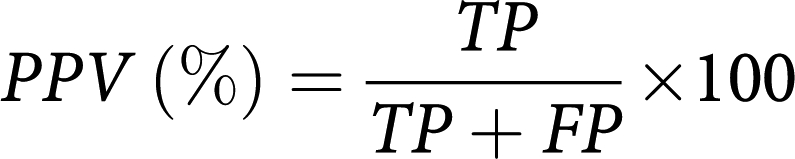


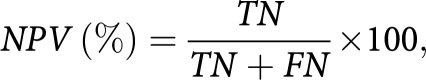
 where TP is the number of true positives, FP the number of false positives, TN the number of true negatives and FN the number of false negatives.

## Results

We have set up a relatively simple and portable system for fluorescence diagnostic imaging. Figure 1 shows the schematic diagram of the setup comprising a fluorescence endoscope interfaced to a PC-based image acquisition and analysis system. This setup enabled us to carry out white light and hypericin fluorescence imaging of lesion and healthy sites in the human oral cavity in a clinical setting. Red fluorescence was observed in lesions, indicating a selective uptake of hypericin in lesions compared with normal tissue.

**Figure 2:**
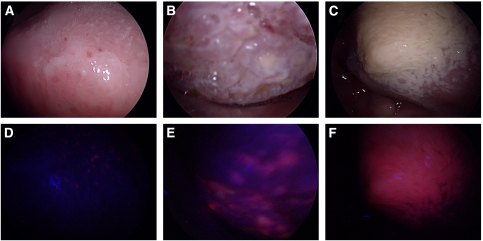
White light (**A–C**) and hypericin fluorescence (**D–F**) endoscopic images of normal (**A**, **D**), hyperplastic (**B**, **E**) and SCC (**C**, **F**) tongues. The fluorescence images show a progressive increase in the red-to-blue intensity ratios from normal (R/B ratio=0.3) to hyperplastic (R/B ratio=1.0) to SCC (R/B ratio=2.0) tissue.

A total of 63 lesion and normal sites from the tongue, buccal mucosa, palate and gingiva were imaged. Figure 2 shows the white light (A, B, C) and hypericin fluorescence (D, E, F) endoscopic images of normal (A, D), hyperplastic (B, E) and SCC (C, F) tongues. The fluorescence images show a progressive increase in the red-to-blue intensity ratios from normal (R/B=0.3) to hyperplastic (R/B=1.0) to SCC (R/B=2.0) tissue.

**Figure 3:**
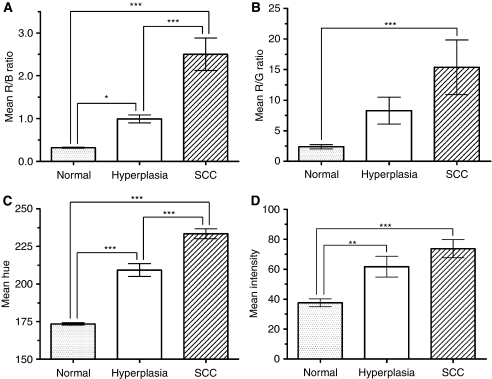
Bar charts showing the mean values of the red-to-blue intensity ratios (**A**), red-to-green intensity ratios (**B**), image hue (**C**) and image intensity (**D**) in normal (*n*=28), hyperplastic (*n*=10) and SCC (*n*=12) oral cavity tissue. Error bars are the s.e.m. Data were analysed using one-way ANOVA with Bonferroni's multiple comparison tests. Asterisks over the respective data pair indicate significant *P*-values (^*^P<0.05; ^**^P<0.01; ^***^P<0.001).

The image parameters, hue, saturation and intensity, as well as the ratiometric parameters, red-to-blue and red-to-green intensity ratios, were extracted and analysed for their ability to distinguish between normal and lesional oral tissue. Figure 3 shows the bar charts of the mean values of the red-to-blue intensity ratios (A), red-to-green intensity ratios (B), image hue (C) and image intensity (D) in normal (*n*=28), hyperplastic (*n*=10) and SCC (*n*=12) oral cavity tissue. A pairwise comparison of the means was carried out using one-way ANOVA with Bonferroni's multiple comparison tests. A *P*-value of less than 0.05 is considered significant and is indicated by asterisks over the respective data pair (^*^P<0.05; ^**^P<0.01; ^***^P<0.001). The results show that the red-to-blue intensity ratio and the image hue can best distinguish between normal, hyperplastic and SCC oral tissue. The red-to-green intensity ratio did reasonably well in distinguishing between normal and SCC tissue, but was not good for distinguishing between normal and hyperplastic tissue and between hyperplasia and SCC. The image intensity was good for distinguishing between normal and hyperplastic tissue and between normal and SCC tissue, but not for distinguishing between hyperplasia and SCC. The image saturation was a poor test parameter overall and the results are not shown.

The above test parameters were also analysed using the ROC curves method to check the quality of the test and obtain the cutoff values for different levels of sensitivity *vs* specificity. The best trade-off value offering the highest combined sensitivity and specificity was chosen for the test parameter between each pair of normal, hyperplastic and SCC data. Tables 1,2,3 show the ROC results and the PPV and NPV for the test between (1) normal and hyperplastic, (2) normal and SCC and (3) hyperplastic and SCC oral tissue, respectively. For distinguishing between normal and hyperplastic oral tissue, the red-to-blue (R/B) ratio and image hue are good test parameters offering 96 and 100% specificity, respectively, at a 100% sensitivity level. Similarly, for distinguishing between normal and SCC tissue, the R/B ratio and hue are good tests, both offering 100% specificity at a 100% sensitivity level. For distinguishing between hyperplasia and SCC, the R/B ratio and hue offer the best results at a specificity of 90 and 80%, respectively, at a 92% sensitivity level.

Overall, the R/B ratio and hue are the best test parameters, giving the best distinction between normal, hyperplastic and SCC oral tissue. The red-to-green intensity ratio and image intensity serve reasonably well to distinguish between normal and hyperplastic and normal and SCC tissue, but distinguish poorly between hyperplastic and SCC tissue. The image saturation value is overall a poor discriminator of oral tissue type and the results are not shown.

## Discussion

Fluorescence imaging of the human oral cavity using hypericin has great potential as a complementary technique to standard white light examination and histopathology of oral cavity lesions. Red fluorescence was observed in lesions, indicating a selective uptake of hypericin in lesions compared with normal tissue. Moreover, hyperplastic and SCC lesions show different levels of fluorescence.

As in our previous studies, we present the red-to-blue (R/B) intensity ratio rather than the red intensity alone, because taking the ratio of the emitted red signal to a portion of the blue excitation light helps eliminate variations due to day-to-day fluctuations of the excitation source strength and geometrical factors such as probe-to-tissue distance (Zheng *et al*, 2002). Thus, the R/B ratio can be thought of as the red fluorescence normalised to the blue excitation light. This ratio was observed to increase progressively from normal to hyperplastic to SCC oral tissue, and is to be expected, as lesions tend to retain drugs for a longer period of time compared with normal tissue. At 2 hours after topical application, most normal tissues will have cleared hypericin, whereas lesions still show fluorescence. SCC tissue is likely to be partially ulcerated and heavily inflamed. Highly vascular tissue is likely to be close to the tissue surface, resulting in brighter fluorescence compared with normal tissue. As shown in our results, the R/B ratio is a good image parameter for distinguishing between normal, hyperplastic and SCC tissue.

As hypericin fluoresces in the orange–red region (emission peak about 590 nm), the green fluorescence emitted is most likely because of autofluorescence of the tissue. The green-to-blue (G/B) intensity ratio, which can be thought of as autofluorescence normalised to the blue excitation light, showed no particular trend between normal, hyperplastic and SCC tissue (results not shown here). Thus, the G/B ratio may not be a good image parameter for distinguishing tissue types. On the other hand, the red-to-green (R/G) intensity ratio, which can be thought of as a ratio of tissue fluorescence to autofluorescence, could distinguish between normal and hyperplastic tissue and between normal and SCC tissue reasonably well in our study.

Among the patient pool, several special cases were excluded from the ROC analysis and will be discussed here. The first case is that of a patient who had undergone glossectomy and was diagnosed as having hyperplasia of the floor of the mouth. Images taken from the lesion showed low fluorescence and were dark, whereas images taken from a normal part of the oral cavity in the same patient were within the normal range. We theorise that in post-surgery tissue, the epithelium and vasculature may not have recovered, resulting in low fluorescence, and the tissue may also have low reflectivity, resulting in dark images.

A patient who had hyperplasia of the tongue with a histologically confirmed presence of Candida was also excluded from the ROC analysis. Candida is known to take up fluorescent dyes, possibly resulting in a false positive (al-Bagieh, 1991). The fluorescence images captured had R/B ratios that were higher than the mean R/B value of hyperplastic lesions in our study.

Another case of interest was that of a patient with cellular pleomorphic adenoma of the palate. As adenomas are glandular in nature, they may accumulate a higher level of photosensitiser, thus giving a higher level of fluorescence than usual. The fluorescence images analysed yielded R/B ratios that were closer to those of SCC than of benign hyperplastic lesions.

Finally, it is worthwhile to discuss where dysplasia fits in our current set of results. As there were only four cases of dysplasia in our patient pool, and this number is too small to form a category, they were excluded from the ROC analysis. From the limited number of images analysed, it seems that the red fluorescence level in dysplasia lies somewhere in the upper half of the range of hyperplasia fluorescence. It could thus be challenging to use fluorescence imaging to distinguish dysplasia from hyperplasia and SCC, but more data need to be collected to confirm this.

Although these special cases indicate the possible limitations of fluorescence imaging in the diagnosis and staging of oral lesions, the technique remains promising. Currently, image analysis takes place offline and a digital diagnosis is determined only after patient visit. Further development of a real-time image acquisition and analysis system interfaced to the fluorescence endoscope will allow acquired images to be analysed on the spot within automatic or user-selected regions of interest to extract image parameters. These image parameters can then be compared to pre-defined threshold levels to determine whether the tissue is normal, hyperplastic or malignant. This will enable same-day diagnosis and cancer staging to be achieved in a clinical setting and provide a visual aid for demarcation of tumour margins, thus enhancing the clinical usefulness of fluorescence imaging.

In conclusion, hypericin fluorescence imaging is promising as a complementary technique to white light endoscopy of human oral cancers by facilitating guided biopsies in the clinic. This may reduce the number of biopsies taken, enable delineation of tumour margins during surgical procedures and provide a means for same-day diagnosis in the clinic.

## Figures and Tables

**Table 1:** Performance indicators of image parameters, the mean red to blue (R/B) and red to green (R/G) intensity ratios, and the mean image hue and intensity values, used to discriminate between normal (*n*=28) and hyperplastic (*n*=10) oral tissue

**Image parameter**	**Area under ROC curve (±s.e.)**	***P*-value**	**Cutoff value**	**Sensitivity (%)**	**Specificity (%)**	**PPV (%)**	**NPV (%)**
Mean R/B	0.9964 (±0.006)	<0.0001	>0.422	100	96	91	100
Mean R/G	0.9071 (±0.05)	0.0002	>2.435	100	75	59	100
Mean hue	1.0 (±0.0)	<0.0001	>183.5	100	100	100	100
Mean intensity	0.8286 (±0.07)	0.002	>41.56	100	64	50	100

**Table 2:** Performance indicators of image parameters, the mean red to blue (R/B) and red to green (R/G) intensity ratios, and the mean image hue and intensity values, used to discriminate between normal (*n*=28) and SCC (*n*=12) oral tissue

**Image parameter**	**Area under ROC curve (±s.e.)**	***P*-value**	**Cutoff value**	**Sensitivity (%)**	**Specificity (%)**	**PPV (%)**	**NPV (%)**
Mean R/B	1.0 (±0.0)	<0.0001	>0.740	100	100	100	100
Mean R/G	0.9464 (±0.03)	0.0002	>2.509	100	75	63	100
Mean hue	1.0 (±0.0)	<0.0001	>198.0	100	100	100	100
Mean intensity	0.9554 (±0.03)	<0.0001	>45.31	100	71	60	100

**Table 3:** Performance indicators of image parameters, the mean red to blue (R/B) and red to green (R/G) intensity ratios, and the mean image hue and intensity values, used to discriminate between hyperplastic (*n*=10) and SCC (*n*=12) oral lesions

**Image parameter**	**Area under ROC curve (±s.e.)**	***P*-value**	**Cutoff value**	**Sensitivity (%)**	**Specificity (%)**	**PPV (%)**	**NPV (%)**
Mean R/B	0.9250 (±0.06)	0.0008	>1.231	92	90	92	90
Mean R/G	0.6333 (±0.12)	0.3	>6.659	75	60	69	67
Mean hue	0.9083 (±0.06)	0.001	>219.5	92	80	85	89
Mean intensity	0.7000 (±0.12)	0.1	>53.75	92	60	73	86
